# Study of the Anti-Staphylococcal Potential of Honeys Produced in Northern Poland

**DOI:** 10.3390/molecules23020260

**Published:** 2018-01-28

**Authors:** Katarzyna Grecka, Piotr M. Kuś, Randy W. Worobo, Piotr Szweda

**Affiliations:** 1 Department of Pharmaceutical Technology and Biochemistry, Faculty of Chemistry, Gdańsk University of Technology, ul. G. Narutowicza 11/12, 80-233 Gdańsk, Poland; kagrecka@gmail.com; 2 Department of Pharmacognosy, Wrocław Medical University, ul. Borowska 211a, 50-556 Wrocław, Poland; kus.piotrek@gmail.com; 3 Department of Food Science, Cornell University, Ithaca, NY 14853, USA; rww8@cornell.edu

**Keywords:** honey, antibacterial activity, biofilm, *Staphylococcus*, antioxidants, phenolics, correlations, MIC

## Abstract

The antimicrobial activity of 144 samples of honeys including 95 products from apiaries located in Northern Poland was evaluated. The antibacterial activity of those natural products, their thermal stability, and activity in the presence of catalase was investigated by microdilution assays in titration plates. The MTT assay was performed for the determination of anti-biofilm activity. Spectrophotometric assays were used for the determination of antioxidant potential, total phenolic content, and ability to generate hydrogen peroxide. Some of the investigated honeys exhibited surprisingly high antimicrobial, especially anti-staphylococcal, potential, with Minimal Inhibitory Concentration (MIC) values of only 1.56% (*v/v*). Much higher resistance was observed in the case of staphylococci growing as biofilms. Lower concentrations of the product, up to 12.5% (*v/v*) stimulated its growth and effective eradication of biofilm required concentration of at least 25% (*v/v*). Hydrogen peroxide has been identified as a crucial contributor to the antimicrobial activity of honeys supplied by Polish beekeepers. However, some of the results suggest that phytochemicals, especially polyphenols, play an important role depending on botanical source (both positive, e.g., in the case of buckwheat honeys as well as negative, e.g., in the case of some rapeseed honeys) in their antimicrobial potential.

## 1. Introduction

For centuries honey was not only a popular sweet component of the human diet, but was also one of the most important drugs used in folk medicine [1]. Regular consumption of this product provides a number of health benefits and its therapeutic potential depends on the botanical origin of the nectar that is used for production of the honey. For instance, in Polish folk medicine, heather (*Calluna vulgaris* L.) honey is proposed as a remedy for prostate, liver and biliary system diseases. Honey collected from rapeseed (*Brassica napus* L.) is recommended for patients suffering from cardiovascular system diseases. Consumption of buckwheat (*Fagopyrum esculentum* Moench) honey is beneficial in relieving the symptoms of hypertension and atherosclerosis [2]. Moreover, honey is used in treatment of infectious diseases, especially difficult-to-heal infected wounds [1,3]. Numerous studies confirm that honey derived from many different botanical and geographical origins exhibit inhibitory effect towards a broad spectrum of Gram-positive and Gram-negative pathogenic bacteria, including antibiotic resistant strains [4,5,6,7,8,9,10]. The antimicrobial action of honey is based on several mechanisms: the acidity (low pH) [5,11,12], osmotic pressure (high sugar concentration) [5,13] and the presence of bacteriostatic and bactericidal factors such as hydrogen peroxide [14,15,16], phenolics [17], peptide—bee defensin-1 [5,18,19], methylglyoxal [20,21] and Maillard reaction products [22]. The Worobo group also found that honey should be considered as a potential source of microorganisms producing promising antimicrobial compounds, especially bacteriocins of a broad bactericidal spectrum [23]. Among all these mechanisms, enzymatic production of H_2_O_2_ is absolutely crucial for antimicrobial activity of the majority of investigated honey types [5,8,12,20]. Reports of several authors revealed that treatment of honey samples with high temperature and/or catalase completely reduces antimicrobial activity of these products [5,9,14,24]. In fact, only in the case of New Zealand’s manuka honey, and several Australian and Malaysian honeys, high level of antibacterial activity is mostly caused by a non-peroxide component—methylglyoxal [21,25,26]. The role of phytochemicals in the activity of honeys produced from nectar of other plants remains unclear. However, some important findings in this area have been recently provided by the Brudzynski and coworkers. Within the group of six honeys (excluding manuka honey) they observed an important correlation between high antioxidant and antimicrobial activity of products and lack of activity in the case of products pre-treated with catalase. As an explanation of this observation, they discovered that honeys containing a high concentration of polyphenols of high radical scavenging activity efficiently produce hydroxyl radicals, which are crucial for the antimicrobial potential of the product. The hydroxyl radicals are produced using H_2_O_2_ (generated by glucose oxidase that is present in honey) as a substrate in the polyphenol-mediated Fenton-like reaction [7,24,27]. As it was shown previously by the groups of Cao [28] and Sakihama [29], the efficiency of this reaction increases dramatically in the presence of polyphenols. Thus, polyphenols present in honey emerge as active intermediates that are necessary to confer oxidative action of hydrogen peroxide. The generated OH˙ radicals are powerful but short-lived oxidants that cause proteins and lipid peroxidation and DNA and RNA degradation in bacterial cells [7,24,27]. However, it also should be noted that many reports have not confirmed the correlation between total phenolic content or antioxidant activity and antimicrobial potential of the product [30,31]. Thus, in our opinion, the crucial role of polyphenols in the generation of OH˙ radicals is not a complete explanation of the role of phytochemicals in the antimicrobial activity of all hydrogen peroxide dependent honeys. The Brudzynski group also revealed that the two-phase colloidal system, consisting of large, micron-size particles distributed in the concentrated sugar solution, is required for antibacterial activity of honey. The enzyme glucose oxidase efficiently produces the hydrogen peroxide only in the situation when it is involved in the structure of these particles which also contain complexes of other proteins, polyphenols and oligosaccharides. The authors found that dilution of honey allows unpacking and dissociation of large, micron-size, superstructures into smaller nanosize particles. The phase transition (destruction of large particles) ensues at the threshold concentration of molecular crowders (glucose and fructose). The glucose oxidases released from the particles is much less active and the phase transition point is critical for antimicrobial potential of honeys [32].

The main scope of the current study was: (1) to investigate antibacterial activity of various types of Polish honeys and their mechanism; (2) to investigate anti-staphylococcal activity (against both reference strains and clinical isolates) of honey solutions against planktonic cells and biofilm as well as to compare MIC, MBC and MBEC_50_; (3) to investigate the role of phytochemicals in H_2_O_2_ mediated antibacterial activity of the products.

## 2. Results

### 2.1. Investigation of Antimicrobial Potential of Honey Samples—Determination of MIC (Minimum Inhibitory Concentration) and MBC (Minimum Bactericidal Concentration) Parameters

The research revealed that some of the investigated honeys exhibited surprisingly high antimicrobial, especially anti-staphylococcal, potential (Table 1). Five of the honeys, all collected by beekeepers from the region of north Poland, were able to inhibit the growth of *S. aureus* reference strains at concentration of only 1.56% (*v/v*). The same MIC value was obtained for seven honeys in the case of *S. epidermidis.* These honeys were two-fold more effective against staphylococci in comparison to reference sample of manuka honey containing high concentration of MGO (methylglyoxal) (at least 550 mg/kg). Thirty two honeys exhibited activity comparable to manuka honey (MIC = 3.125% *v/v*) when tested against *S. aureus* ATCC 25923 and *S. epidermidis* ATCC 12228. The strain *S. aureus* ATCC 29213 exhibited a slightly higher resistance, at 3.125% (*v/v*) its growth was inhibited by twenty six honey samples. On the other hand, a large group of the honey samples did not exhibit anti-staphylococcal activity within the investigated range of concentration (0.39–12.5% (*v/v*)). MIC higher than 12.5% (*v/v*) was observed for forty two, forty five and thirty six honeys, in the case of *S. aureus* ATCC 25923, *S. aureus* ATCC 29213 and *S. epidermidis* ATCC 12228 reference strains, respectively. Gram-negative bacteria, especially *E. coli*, revealed lower sensitivity. Lack of activity within the tested range of concentration (MIC > 12.5%) was obtained for seventy and forty seven samples in the case of *E. coli* and *P. aeruginosa*, respectively. None of the honeys were able to inhibit the growth of these microorganisms at concentrations lower than 3.125% (*v/v*), and this level of activity (MIC = 3.125% (*v/v*)) was obtained for only two honeys in the case of *E. coli* and four samples in the case of *P. aeruginosa.* The MIC values of manuka honey for both these bacteria were 6.25% (*v/v*), and the same activity exhibited eleven and twenty six of honeys from our collection, respectively.

In relation to the sources of the product (local apiary, organic grocery store, supermarket and abroad), we found that the most active honeys were supplied by the beekeepers from the north of Poland. As presented, over twenty seven and twenty two out of ninety five (28.4% and 23.2%) of these honey samples exhibited equal or higher activity compared to that of manuka honey against *S. aureus* ATCC 25923 and *S. aureus* ATCC 29213 strains, respectively. However, in this group, a large number of honey samples (twenty three and twenty seven against ATCC 25923 and ATCC 29213 strains, respectively) did not exhibit activity within the investigated range of concentration (MIC > 12.5%). Satisfactory activity was also observed for honeys coming from sources abroad (*n* = 14, excluding manuka honey). Five of the foreign honey samples (35.7%) inhibited the growth of both *S. aureus* reference strains at concentrations equal to the activity of manuka honey (3.125% (*v/v*)). Honeys purchased in supermarkets, and especially in organic grocery stores revealed a bit lower activity, with a high percentage of non-active samples (41.7% and 45.4%, respectively—in relation to both *S. aureus* strains). Some interesting conclusions come also from the analysis of the dependence of activity in relation to the botanical source of honey, especially in the case of products collected from *Fagopyrum esculentum* Moench and *Tilia* spp.. Among eleven samples of buckwheat honey supplied by beekeepers, three (27.3%) and four (36.4%) exhibited activity higher than manuka honey against both *S. aureus* and *S. epidermidis* reference strains respectively, with MIC values of 1.56% (*v/v*). Three of them revealed also higher activity against *P. aeruginosa* ATCC 27853 with MIC value of 3.125%. Activity equal (MIC = 6.25%; *n* = 4); or better (MIC = 3.125%; *n* = 1) than Man550 (manuka honey, the number corresponds to minimal MGO content in mg/kg) was also observed against *E. coli* ATCC 25922*.* Only one sample (9.1%) of buckwheat honey supplied by beekeepers was not able to inhibit the growth of any of the three reference staphylococci, and one more sample did not inhibit *S. aureus* ATCC 29213 within the investigated range of concentrations. Two and five samples (18.2% and 45.4%) were not active against *P. aeruginosa* and *E. coli*, respectively*.* Honeys produced from the same botanical source, but purchased in supermarkets and especially in organic grocery stores, did not exhibit promising activity. The lime honeys (*n* = 11) also exhibited high activity mostly against *Staphylococcus* spp.. The MIC values for seven lime honeys (63.6%) were ≤ 6.25% (*v/v*) against all reference staphylococci, which should be classified as a satisfactory result. Contrary to buckwheat honey, dependence between the activity and the source of the product was not observed, as honeys supplied by beekeepers and purchased in shops presented similar antibacterial potential. Comparable results were obtained in the case of honeydew honey. The MIC values against staphylococci for six out of ten investigated samples (60.0%) were 3.125 or 6.25% (*v/v*). Four following samples did not exhibit activity and the dependence between activity and the source from where the honeys were supplied were not observed. Much lower activity was observed in the case of honey derived from the nectar of rapeseed (*Brassica napus* L). At the concentration of 12.5% (*v/v*), three out of seven (42.9%) samples were not able to inhibit the growth of any of the reference strains of bacteria, and only two samples inhibited the growth of *S. aureus* ATCC 25923 at concentration equal to 6.25% (*v/v*) and only one of the samples was effective at this concentration against *S. epidermidis* ATCC 12228. Only singular samples were available for our research in the case of other honeys with declared botanical sources. Their activity was rather low or medium with MIC values not lower than 6.25% (*v/v*). Interestingly, some multi-floral honeys, especially in the case of samples obtained from local beekeepers (*n* = 64), including products collected from forest and peatland areas exhibited good antimicrobial activity. Two (3.1%) of these honeys were classified as most active against staphylococci with MIC = 1.56% (*v/v*) and one further honey exhibited very high activity, but only against *S. epidermidis* ATCC 12228*.* Moreover, sixteen (25%), thirteen (20.3%) and eighteen (28.1%) samples of this group of honeys inhibited the growth of *S. aureus* ATCC 25923, *S. aureus* ATCC 29213, and *S. epidermidis* ATCC 12228, respectively, at a concentration of 3.125% (*v/v*). This correlates to the same activity as manuka honey. High activity was also revealed for the group of ten honeys, of undefined botanical source, coming from abroad. With the exception of one, all of them inhibited the growth of all *Staphylococcus* spp. reference strains at a concentration of 3.125% or 6.25% (*v/v*). Multi-floral honeys coming from market sources exhibited lower effectiveness. For many honeys, bactericidal activity required at least two-fold higher concentration, in comparison to the concentration that guarantees bacteriostatic effect. The shift of concentration of MBC value in comparison to MIC was observed for both Gram-positive and Gram-negative bacteria, and was especially common in the case of *S. aureus* ATCC 25923 and *P. aeruginosa* ATCC 27853 (forty two honeys for both of pathogens, including manuka honey). Further investigation using staphylococcal strains recovered from clinical and animal sources (*n* = 6 in both cases) confirmed high anti-staphylococcal potential of some honeys (Table 2 and Table 3). 

The MIC values for all twelve selected, based on the results of preliminary studies, honey samples were in the range of concentrations from 0.78 to 3.125% (*v/v*) against all strains tested. Surprisingly, some of the honeys, namely 24, 25, 76, 89, 128, 105 and 139, with very low values of MIC, were much less active in killing staphylococci. It was observed that for some honey samples, they were not able to eliminate the living cells of bacteria up to a concentration of 12.5% (*v/v*). On the other hand, honeys with code numbers 35, 81, 83, 86 and 108 were very effective in both inhibition and killing the cells of all strains tested.

### 2.2. Time-Kill Assay, Determination of Kinetic of Bactericidal Effect of Honey Against Staphylococci

**Figure 1:**
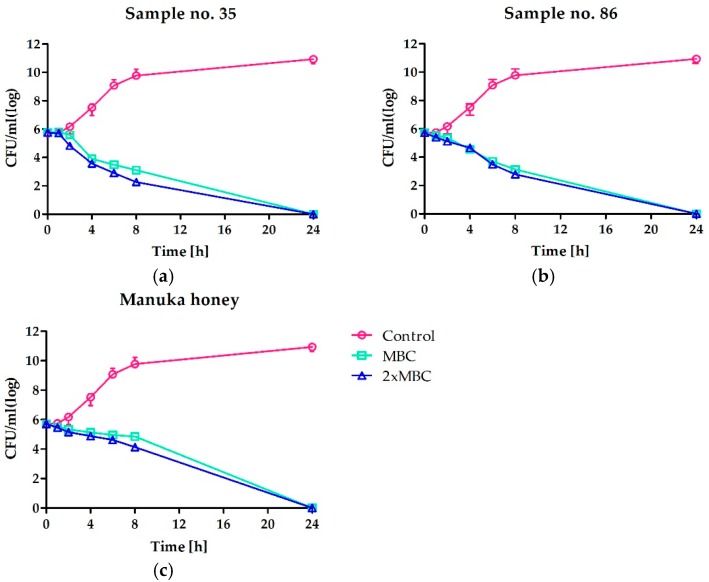
Time-kill curves of *S. aureus* ATCC 25923 cultivated in absence or presence of two the most active honeys: (**a**) multi-floral honey and (**b**) buckwheat honey, both collected by professional beekeepers; in comparison to manuka honey (**c**). Concentrations of honeys are presented in relation to MBC determined.

The time-kill assay kinetics additionally confirmed the bactericidal potential of the most active honeys against staphylococci (Figure 1). However, the complete elimination of living cells of *S. aureus* ATCC 25923 required extended incubation with honeys (both Polish honeys and Man550 honey) up to 24 h. Nearly the same effect was observed when the honeys were used at the concentration equal to the MBC value or 2 × MBC. Interestingly, both honeys collected in Polish apiaries (assigned as 35 and 86) revealed slightly higher activity in comparison to Man550 during the first eight hours of incubation. In the case of both honeys, the population of the reference strain was reduced by about 4 log (CFU/mL), whilst treatment with manuka honey resulted in reduction of the number of bacteria by about 2 log (CFU/mL).

### 2.3. Activity of Honeys against Staphylococci Growing in the Form of Biofilm

**Figure 2:**
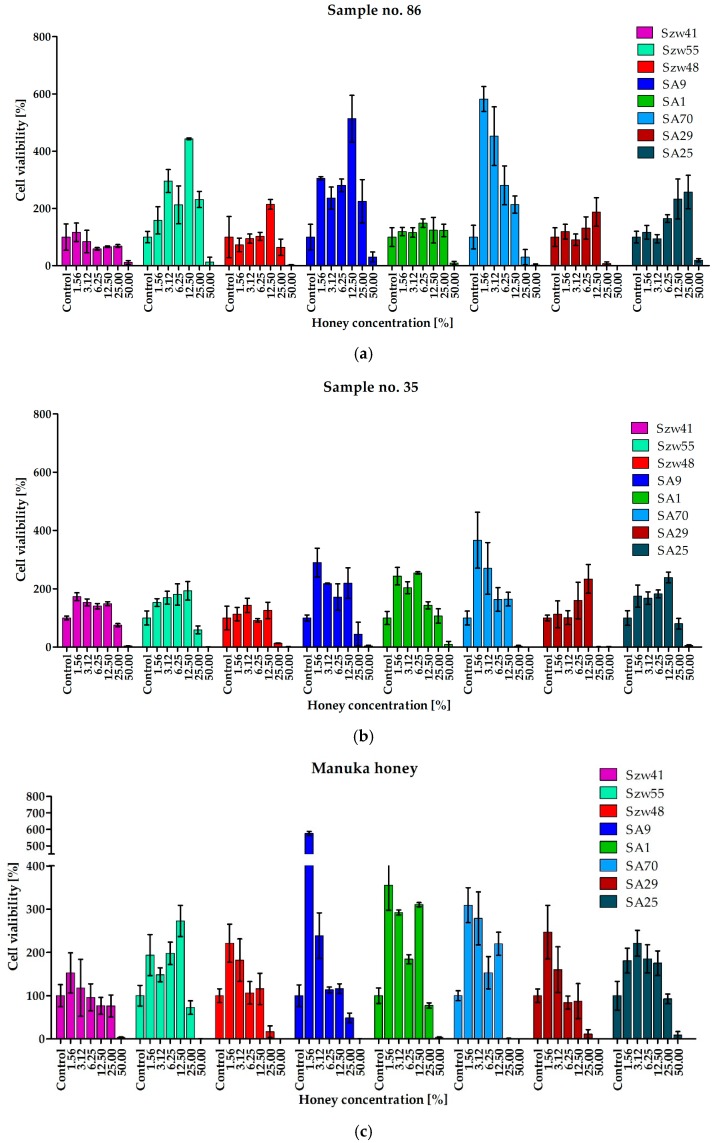
Anti-biofilm activity of selected honeys: (**a**) 86; (**b**) 35; (**c**) manuka.

In clinical scenarios, staphylococci that are responsible for infections often exist in the form of biofilms. Bacterial biofilms, including *Staphylococcus* spp., are characterized by exhibiting extremely high resistance to most antimicrobial agents, including antibiotics. [33,34]. The research presented here revealed that the increased resistance of staphylococcal biofilms in comparison to planktonic cells, was also observed with the sensitivity to the antimicrobial activity of honey. Even in the case of Man550 and the most active honeys from our collection, with MIC values against planktonic cells as low as 1.56% (*v/v*), inhibition of biofilm formation required concentration of at least 25% (*v/v*). For most strains, complete eradication of biofilm was achieved at the concentration of 50% (*v/v*) (Figure 2; Table 4).

Another important observation from this part of the research is the fact that the presence of honey at a concentration lower than MBEC_50_ value significantly stimulates the growth of the biofilm (Figure 2). In the media containing honey at a concentration below the MBEC_50_ values, up to six-fold higher intensity of biofilm growth was observed with some strains (e.g., strain SA9 treated with honeys 86 and Man550 at concentrations of 12.5 and 1.56% (*v/v*), respectively).

### 2.4. Determination of Mechanisms of Antimicrobial Activity of Investigated Honeys

Heat and catalase treatment confirmed that enzymatic generation of hydrogen peroxide is absolutely crucial for antimicrobial activity of honeys delivered by beekeepers from north of Poland. Only one honey (No. 35) exhibited residual activity (MIC = 12.5%) in the medium containing catalase. All honeys selected for this assay completely lost their activity in the consequence of only 10 min incubation at 80 °C. Twenty minute incubation at 60 °C resulted in complete loss of activity of seven samples, and in the case of five remaining samples, there was a decrease of activity observed. In fact, even 10 min incubation at 60 °C was sufficient for important decrease of activity of all honeys except one (Table 5).

**Figure 3:**
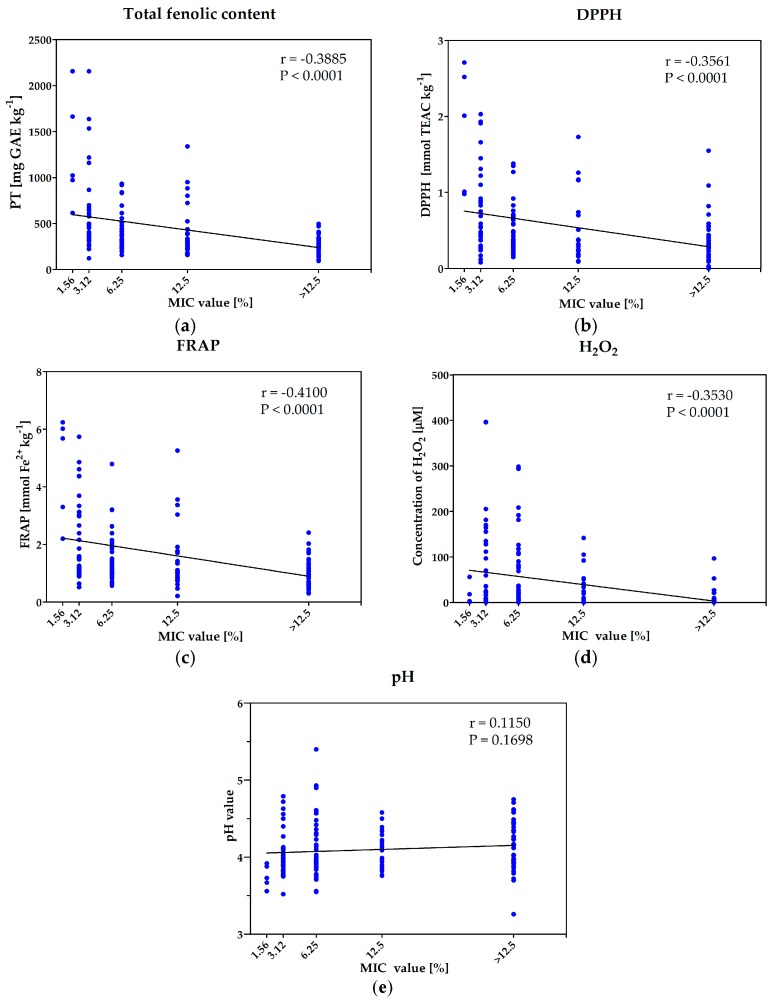
Results of statistical analyses of correlation between antimicrobial activity and some of the investigated parameters: (**a**) total phenolic content; (**b**) antioxidative properties determined with DPPH assay; (**c**) antioxidative properties determined with FRAP assay; (**d**) concentration of generated hydrogen peroxide; (**e**) acidity—pH value.

The investigated samples showed large variability in terms of antioxidative potential (FRAP and DPPH) and total content of phenolic ingredients. The phenolic content ranged from 93.0 to 2156.7 mg GAE/kg and was the lowest in black locust honey (sample No. 101), and the highest with buckwheat honeys (sample No. 86). The antioxidant activity ranged from 0.0 to 2.7 TEAC mmol/kg in DPPH assay, and from 0.2 to 6.2 mmol Fe^2+^/kg in FRAP assay, and was the highest in multi-floral product (sample No. 35) and the lowest in black locust (sample No. 104). The statistical analysis revealed significant, negative, correlation between the concentration of phytochemicals (polyphenols and antioxidants) and MIC values of the honeys (higher concentration of polyphenols as well as higher antioxidative potential resulted in lower value of MIC) (Figure 3a–c). Thus, the concentration of phytochemicals is positively correlated with antimicrobial potential of honeys against *S. aureus* ATCC 29523 strain (MIC is negatively correlated with antimicrobial activity of the products). However, more detailed analysis of the results is required. In fact, each sample should be considered individually. Five of the most active honeys (MIC = 1.56% *v/v*) were characterized with very high concentrations of polyphenols and antioxidative potential (both assays), which is in agreement with the result of statistical analyses. However, it also should be noted that many honey samples with MIC = 0.312 or MIC = 0.625 % (*v/v*), were characterized with low values of parameters determined in DPPH and FRAP assays as well as low concentration of phenolic ingredients.

The simple statistical analyses carried out for the whole group of samples could also lead to the wrong conclusions in the case of determination of amount of hydrogen peroxide generated in the diluted honeys. Similarly, as in the case of antioxidant activity/polyphenols content, significant dependence of antimicrobial potential and concentration of generated H_2_O_2_ was revealed (Figure 3d). However, all honeys with the lowest MIC value (1.56% (*v/v*)) were characterized with low concentrations of hydrogen peroxide. Similar low concentration of this agent was also identified in many products with MIC values equal to 3.125% or 6.25% (*v/v*). In agreement with the results of statistical analyses, many products with low antibacterial activity contained low concentration of H_2_O_2_ (however, not lower than many honeys with high antimicrobial potential). Some individual samples of low activity (MIC = 12.5% (*v/v*)) or even non-active products generated a quite high concentration of H_2_O_2_—of about 100 μM. These results are in contradiction to previous observations, that enzymatic generation of hydrogen peroxide is crucial for antimicrobial activity of Polish honeys (treatment with catalase and high temperature). To solve this problem additional investigation, aiming at the enzymatic generation of hydrogen peroxide in honey solutions (25% *v/v*) prepared in H_2_O_2_ (100 μM) solution used as a solvent (instead of water), was carried out.

**Figure 4:**
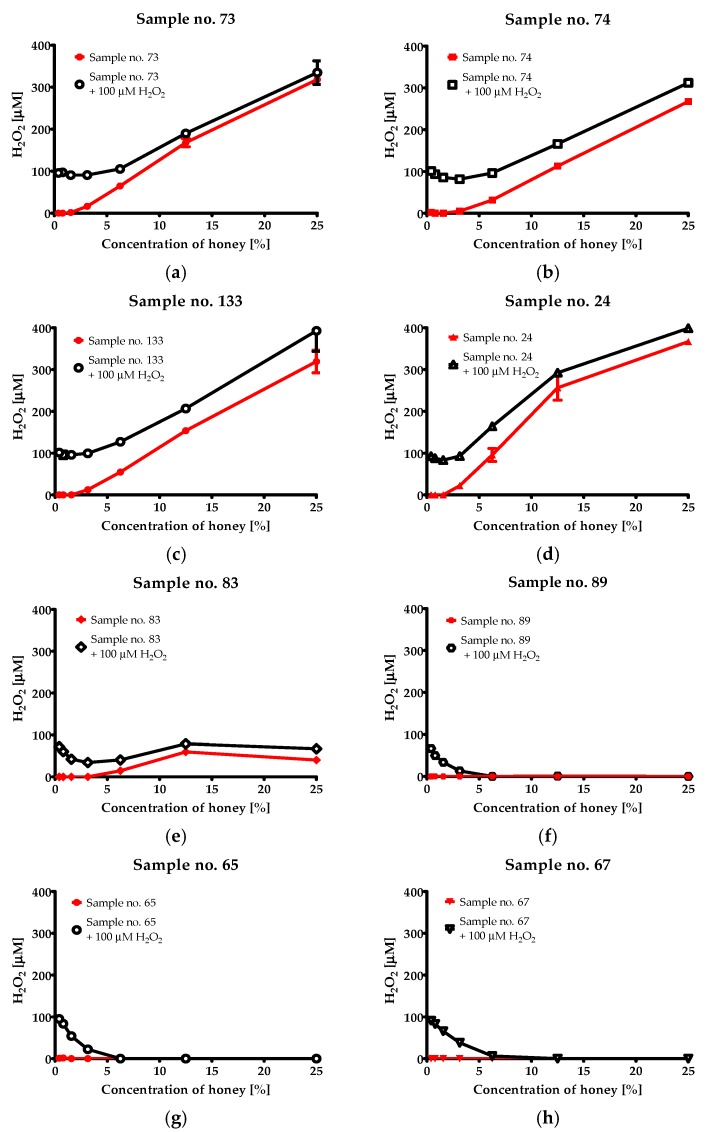
Investigation of hydrogen peroxide concentrations in honey solutions prepared in 100 μM H_2_O_2_ as a solvent; (**a**,**b**) lime tree (**c**,**d**) multi-floral (**e**,**f**) buckwheat (**g**,**h**) rape honey.

The suspected summing up of H_2_O_2_ concentrations (coming from solvent and generated by glucose oxidase) was observed in the case of some multi-floral (Figure 4c,d), honeydew (not shown) and especially lime tree honeys (Figure 4a,b). Surprisingly, important or even complete decomposition of H_2_O_2_ was observed in the consequence of addition of even low concentration of buckwheat honeys (recognized as most active with MIC values of 1.56% or 3.125% (*v/v*)) (Figure 4e,f) as well as honeys produced from rapeseed nectar (not active against bacteria) (Figure 4g,h).

The water content of all products was below 20% which is in agreement with the requirements of EU law, and guarantee microbial stability of the product during long time storage. The honey is known as an acidic product. As it was suspected, within the group of interest the pH value ranged from 3.26 to 5.40 and was the lowest in classified as honeydew honey sample (No. 110, bought in organic grocery store) and the highest in multi-floral product assigned with No. 37. However, all products with MIC = 1.56% (*v/v*) (against *S. aureus* ATCC 25923) characterized with low values of pH, the statistical analyses performed for the whole collection of honeys revealed the lack of correlation between acidity and antimicrobial potential of products (Figure 3f).

## 3. Discussion

The research presented here revealed important differences in the antimicrobial potential of honeys produced by Polish beekeepers. Several products, five in the case of *S. aureus* and seven in the case of *S. epidermidis*, exhibited high antimicrobial activity with MIC values of only 1.56% (*v/v*). On the other hand, within the tested range of concentrations from 0.39 to 12.5% (*v/v*), quite a large number of honey samples did not exhibit any activity against the reference strains tested. Similar differentiation of activity was found in our previous research, when honeys from the south of Poland were tested [9], but other authors also observed important differences in the activities of their honey samples from different geographical regions [6,8,24,25]. Gram-negative pathogens *E. coli* and *P. aeruginosa* exhibited lower susceptibility to the honeys’ components. Usually 2 or 4 fold higher concentration was necessary to achieve inhibitory/bactericidal effects in comparison to staphylococci. It is probably a consequence of differences in cell wall construction of these bacteria. Higher susceptibility of staphylococci in comparison to Gram-negative bacteria was also observed by other authors [6,26,27]. However, in many reports the differences in susceptibility were not so evident [35,36].

Despite the fact that many reports concerning antibacterial, including anti-staphylococcal, effectiveness of honeys collected in different regions of the world have been published, such a high level of activity (MIC = 1.56% (*v/v*)) has been rarely observed. The highest anti-staphylococcal activity of honey has been reported by Nishio and coworkers for products collected by stingless bees *Scaptotrigona bipunctata* (MIC = 0.62% or 1.25% depending on the *S. aureus* strain tested) and *Scaptotrigona postica* (MIC = 1.25% or 2.5% depending on the *S. aureus* strain tested) [37]. High effectiveness (MIC < 2%) against *S. aureus* (including MRSA) was confirmed for Scottish heather honey [38], and chestnut, fir and forest honeys from Slovenia inhibited the growth of *S. aureus* at concentration of 2.5% (*v/v*) [8]. MIC values of about 3.0% (*v/v*) were observed in the case of e.g., Chilean ulmo tree honey [39], some Greek and Cypriot honeys [6], Canadian buckwheat honey [27] and some Polish honeys investigated in our previous report [9]. Comparable activity has been also confirmed for honey produced from the manuka bush (*Leptospermum scoparium*) indigenous to New Zealand and Australia [21,25]. Interesting results have been recently presented by Alvarez-Suarez and coworkers [40]. The authors found some important differences in properties, including antimicrobial potential, between the Cuban multi-floral honeys produced by two different bee species: *Melipona beecheii* and *Apis Mellifera*. In the case of *S. aureus* the honey collected by *M. beecheii* revealed over seven–fold higher activity in comparison to the honey produced by *A. mellifera*, with Minimum Active Dilution (MAD) values of 2.0% and 15.0%, respectively. However, a bit different method was used for determination of antimicrobial activity in this study we have no doubts, that the product collected by *M. beecheii* belongs to honeys with the highest antimicrobial potential described to date [40]. Several other *in vitro* investigations revealed that honeys produced in many other geographical regions e.g., Malaysia [26,41], Thailand [30], Nordic [36], Italy [42] and Africa [43,44,45] are also promising candidates for the treatment of infections caused by staphylococci, including MRSA. The higher values of MBC in comparison to MIC parameter of many products (especially in relation to clinical isolates) identified in our studies, have been also presented by other authors, who compared inhibitory and bactericidal potential of honeys [26,30,36]. This situation is common for many other antimicrobial agents, including some antibiotics.

High potential (MIC ≤ 1.56% (*v/v*)) of selected honeys from our collection was also confirmed for *S. aureus* strains isolated from animals (bovine mastitis) and humans (infected wounds), which is very optimistic from the point of view of potential application of Polish honeys in clinical practice, e.g., as a component of wound dressings. As it was mentioned above, in folk medicine honey was an essential agent used for treatment of difficult-to-heal infected wounds. Its high potential for this purpose has been recently confirmed and discussed in many scientific reports and publications [3,46]. Consequently, several pharmaceutical companies have proposed the dressings containing honey (mostly manuka honey), which are successfully used in treatment of infected wounds. Some authors even suggest the possibility of using honey for treatment of systemic, particularly gastrointestinal infections [47]. On the basis of papers published up to December 2014, Henatsch and coworkers reviewed the possibilities of application of honey in otorhinolaryngology. The authors concluded that this product can be considered as effective (additional) treatment in mucositis, childhood cough, persistent post-infectious cough and after tonsillectomy [48]. On the other hand, there are also several reports that revealed lack of positive effects of honey application for infection treatments. In the trial carried out by Kwakman and coworkers, the medical-grade honey did not affect colonization of the skin at central venous catheter insertion sites in intensive care units patients when applied in addition to standard disinfection with 0.5% chlorhexidine in 70% alcohol [49]. The outcomes of investigation carried out by the mentioned above group of Henatsch revealed that honey eardrops showed a strong in vitro inhibitory activity against all tested strains but did not eradicate *S. aureus* infection in vivo [50]. It also should be emphasized that in vivo infections are often caused by bacteria growing in the form of biofilms. Eradication of this type of infection is especially problematic. Bacterial biofilms are extremely resistant to antibacterial agents including antibiotics and disinfectants. Results of our investigations confirmed much higher resistance of bacterial biofilms in comparison to planktonic cells. The MBEC_50_ for most active products was not lower than 25% (*v/v*). High resistance of bacterial biofilm was also confirmed in recent studies of Garcia-Tenesaca and coworkers. The dilution containing 20.0% of avocado honey eradicated about 60% of performed staphylococcal biofilm, and activity of eucalyptus and rapeseed honeys were even lower with the level of biofilm eradication below 50% [51]. Moreover, we observed that a concentration lower than MBEC_50_ significantly enhanced the growth of the biofilm—honey probably is used as a source of glucose (easily digestible source of carbon). A similar effect was previously presented by Lu and coworkers [52]. These observations can be the explanation of failures of treatment of in vivo infections with honey. For successful therapy, the product would have to be used at a concentration not lower than MBEC value. It is not problematic in the case of infected skin wounds (undiluted honey is used for preparation of wound dressing materials), but in the case of systemic infections achievement of such a high concentration would be a challenge, and in most cases is not achievable.

Taking into account botanical sources of the products, especially high activity honeys were derived from buckwheat. It is in agreement with our previous observation [9] and with results presented by other authors [27,36,53,54]. High antimicrobial potential exhibited also products qualified as honeydew honey and honey produced from the nectar of a lime tree, which also has been presented in some other reports [9,36,54]. Interestingly high activity was observed in the case of some multi-floral honeys whilst in the previous report presented by Kędzia and coworkers [54] most of Polish multi-floral honeys were found as products with low antimicrobial potential.

Another important finding of our investigation is the correlation between activity and the source of product (beekeeper, supermarkets and organic grocery stores). The most active honeys were those provided by local beekeepers. High activity was also observed in many honeys coming from abroad. This observation can be (in our opinion) explained by the result of the assay aiming in determination of influence of higher temperature on the activity of the product. We have found that even 10 min treatment the honeys with 60 °C resulted in decrease/complete loss of activity. Honeys delivered by beekeepers have not been processed since harvesting from the hive. Supermarkets and smaller shops usually buy the honeys from big apiaries or from wholesalers. In both cases the product is stored in large-sized containers (usually barrels). During the storage it crystallizes, which is a natural phenomenon (except of honey derived from acacia). Before delivering to the store, the honey is decrystallized and poured into jars. The “liquefaction” of the product should be performed at temperatures not higher than 40 °C, which is safe for honey (its biological value), but is a time consuming process. If the process is performed at higher temperatures, it is much faster, but unfortunately strongly affects stability of some thermal sensitive ingredients including glucose oxidase and other enzymes. The significant correlation between glucose oxidase activity and honey crystals melting time and temperature has been previously revealed by the Kretavicius group [55]. The presented herein results suggest that some of Polish apiaries/wholesalers perform the decrystallization process at too high temperatures.

High sensitivity to elevated temperatures and catalase unambiguously confirms that enzymatic generation of hydrogen peroxide is crucial for antimicrobial activity of Polish honeys (Table 5). On the other hand, the observed strong positive correlations between phenolic content, antioxidant activity, as well as botanical source, and antibacterial activity suggest that components of plant origin (phytochemicals) also importantly affect the products’ activity. The lack of activity with honeys that have inactivated glucose oxidase indicates that the amount of phytochemicals is too low to exert the antibacterial effect alone, or they are present in the honey in less/non-active form. Similar results have been presented by Brudzynski’s group. The authors performed long term research that mostly explained the detailed mechanism of bactericidal potential of hydrogen peroxide dependent honeys and a role of phytochemicals in bacteria elimination. They found that average content of H_2_O_2_ in honey was over 900-fold lower than that observed in disinfectants and was not sufficient to eliminate the bacteria as the only factor. Thus, they concluded that other components of honey enhance the bactericidal potential of hydrogen peroxide [56]. Further investigation revealed polyphenols as intermediates that are necessary to confer oxidative action of hydrogen peroxide—generation of hydroxyl and phenoxyl radicals (discussed in more detail in the introduction) that are directly responsible for killing microorganisms [7,16,24,27,56]. In their studies, Brudzynski and coworkers mainly used buckwheat honey as a model. Herein, we also investigated the results of the reaction of ingredients of other honeys, namely multi-floral, rape, and lime, with H_2_O_2_. Adding buckwheat and rape honeys to the 100 μM hydrogen peroxide solution resulted in dramatic decrease of concentration of this agent and the amount of eliminated H_2_O_2_ correlated with final concentration of the honey. In the light of results presented by Brudzynski and coworkers, the elimination of H_2_O_2_ in the case of buckwheat honey was rather expected and can be easily explained. Interestingly, a similar effect was observed in the case of, not active, rape honey, which has not been observed to date. Very low or even lack of activity of the product derived from this plant suggests that some ingredients effectively react with H_2_O_2_ (introduced to the solution or generated by the glucose oxidases) degrade it, and the generated products do not exhibit antimicrobial activity (in contrast to derivatives of reaction with components of buckwheat honey). The results of this experiment also explain the low level of concentration of hydrogen peroxide in the solutions of both products (buckwheat and rape honeys). The low antimicrobial activity of rape honey has been revealed in several independent investigations carried out in Poland [9,54,57]. This could suggest that some essential phytochemicals present in this product are responsible for elimination of hydrogen peroxide without generation bacteriostatic/bactericidal products.

An explanation of the high activities of multi-floral and lime tree honeys seems to be a bit more complicated. It could be the consequence of generation and accumulation of high concentration of hydrogen peroxide in these products. As it was shown in Figure 4a–d, enzymatically produced H_2_O_2_ is not strongly affected by phytochemicals present in all four samples of these honeys. In all cases we observed summing up of the hydrogen peroxide introduced to the solution and enzymatically produced one. However, in our opinion the complete understanding of mechanisms of elimination of bacteria by these products, especially the role of phytochemicals requires further research. Thus, the issue of influence of phytochemical components to the antimicrobial activity of honey seems to remain unresolved and quite complicated problem, which cannot be explained by one universal mechanism.

The observed high activity of some Polish honeys with MIC values of 1.56% (*v/v*) seems to be also interesting from the point of view of the global structure of honey—the mechanisms stabilizing the structures of micron-size particles, which are crucial for maintaining the glucose oxidase in highly active form. Brudzynski and coworkers proposed concentration of molecular crowders as crucial factors for stabilization of these large-size structures and existence of two-phase coloidal system [32]. The results presented herein suggest that values of phase transition points of some Polish honeys would have to be lower in comparison to the most active products investigated by the Brudzynski group, with a relatively low concentration of essential sugars (up to 4 fold). In our opinion, this result could suggest that the stability of these large-size particles can be strongly dependent on their chemical composition (presence and concentration of some ingredients). Verification of this thesis and identification of key components will be the issue of our future research.

Because of the climate conditions (cold weather, short season when the bees can collect honey and pollen) and the fact that there are not many places where honey plants (e.g., phacelia, melilot or even rape) are grown the beekeeping is not easy in the region where the samples were collected (north of Poland, near Gdańsk). Moreover, definitely most of local beekeepers do not travel with bees to the places where some honey plants are grown, and only some of them have an access to the fields of buckwheat (*Fagopyrum esculentum* Moench), rapeseed (*Brassica napus* L.) or parks, alleys or forests with a larger number of black locust (*Robinia pseudoacacia* L.) or lime (*Tilia* spp.) trees. Consequently, mostly multi-floral honey is produced in this region and the yield of production in local apiaries is very low (according to the last report of Polish Bee Association [58] it is one of the lowest in Poland, of just about 10–17 kg of honey per hive). Nevertheless, the multi-floral honey produced in this region is characterized by unique features, especially taste. In our opinion, the fact that bees collect the relatively low amount of nectar from different botanical origins in hard weather and climate conditions is also important for high antimicrobial potential of the local honeys. Collecting nectar from many different plant species (mostly weeds and herbs) guarantees the presence of a high concentration of many different phytochemicals in the final product. During the honey-making process, bees add to the nectar enzymes secreted by their salivary glands and remove the water to the final concentration of no more than 20%. The main goal of water removing as well as adding glucose oxidase and bee defensin-1 is antimicrobial protection of honey. The fact that in this region bees convert the relatively low amount of nectar guarantees the high concentration of both antimicrobial agents coming from their bodies, namely glucose oxidase and defensin-1 in the final product. The carried out research has revealed the important correlation between the antimicrobial efficiency of honeys and the content of phytochemicals and enzymatically generated H_2_O_2_. The high concentration of defensin-1 could be an explanation of high antibacterial efficiency of the products that contain neither a high concentration of polyphenols nor hydrogen peroxide. Valachova and coworkers [19] have revealed some important differences in the concentration of bee defensin-1 in different honey samples. Comparison of concentration of this peptide in local honeys with the products coming from other geographical regions as well as determination of influence of this agent to the whole antimicrobial efficiency of local honeys will be investigated in our group.

## 4. Materials and Methods

### 4.1. Bacterial Strains and Media

In the preliminary studies, antimicrobial activity of all honeys was tested against five reference strains of bacteria: *S. aureus* ATCC 25923, *S. aureus* ATCC 29213, *S. epidermidis* ATCC 12228, *Pseudomonas aeruginosa* ATCC 27853, and *Escherichia coli* ATCC 25922. The anti-staphylococcal potential of selected—most active honeys, was also investigated against six *S. aureus* isolates from patients with skin and soft tissue infections (SSTI)**:** Szw35, Szw41, Szw17, Szw48, Szw55, Szw16 [59] and six *S. aureus* strains derived from subclinical bovine mastitis (SCM) milk samples: SA1, SA9, SA70, SA102, SA103, SA105 [60]. All strains of bacteria were routinely grown on Mueller-Hinton Agar (MHA, Sigma Aldrich, Schnelldorf, Germany) plates. The Minimum Inhibitory Concentration (MIC) was determined using liquid medium—Mueller-Hinton Broth 2 (MHB2, Sigma Aldrich) and for determination of Minimum Bactericidal Concentrations (MBC) the cells were transferred on the Baird Parker Agar plates (Biomaxima, Lublin, Poland).

### 4.2. Chemicals and Reagents

The standard compounds and reagents (all of analytical grade): MTT (3-(4,5-dimethylthiazol-2-yl)-2,5-diphenyltetrazolium bromide), DMSO (dimethyl sulfoxide), H_2_O_2_ (peroxide hydrogen), o-dianisidine dihydrochloride, Peroxidase from horseradish Type VI, ferrous sulphate, gallic acid, Na_2_CO_3_, ferric chloride, 1,1-diphenyl-2-picrylhydrazyl radical (DPPH), 2,4,6- tris(2-pyridyl)-1,3,5-triazine (TPTZ), (±)-6-hydroxy- 2,5,7,8-tetramethylchroman-2-carboxylic acid (Trolox), Folin-Ciocalteu’s reagent, PBS were purchased from Merck (Darmstadt, Germany). Methanol and H_2_SO_4_ were purchased from (POCH, Gliwice, Poland). Ultrapure H_2_O (18.0 MΩ) was obtained with Milli-Q Advantage A10 system (Millipore, Billerica, MA, USA). The absorbance of reaction mixture in Folin-Ciocalteu, DPPH and FRAP assays were measured using a Genesys 20 spectrophotometer (Thermo Scientific, Waltham, MA, USA).

### 4.3. Honey Samples

The studies covered 144 samples of honey (Table 6.). The primary goal of our research was determination of antimicrobial activity of honeys collected in the northern region of Poland. In 2015 and 2016, beekeepers from this region delivered 95 samples. Most of them (*n* = 58; 61%) were classified as multi-floral honeys, 4 (4.2%) were classified as honeydew honeys, 4 (4.2%) honeys were collected in apiaries located in forests, 2 (2.15%) in peatlands and for 27 (28.4 %) samples the beekeepers were able to denominate the leading species of plants that were the source of nectar for bees. Due to some expected differences in activity, honeys bought in organic grocery stores (*n* = 22) and supermarkets (*n* = 12) were investigated. Moreover the antibacterial activities of Polish north region honeys (PNPH) were compared to the activity of honeys coming from abroad (15), including manuka honey—Man550. The manuka honey was kindly supplied by Propharma (Warszawa, Poland). All the honey samples were stored at 4 °C in the dark.

### 4.4. Investigation of Antimicrobial Potential of Honey Samples—Determination of MIC (Minimum Inhibitory Concentration) and MBC (Minimum Bactericidal Concentration) Parameters

The assay was performed as described in our previous publications [9,61] with slight modifications. Briefly, overnight bacterial cultures grown on MHA plates were suspended in PBS (pH 7.4) to the cell density of OD_600_ = 0.132 (equal to 0.5 McFarland turbidity standard)—approximately 1–5 × 10^8^ CFU/mL and in the next step diluted in MHB2 medium at a ratio of 1:150 *v/v* to the final cells concentrations of approximately 1.5 × 10^6^ CFU/mL.

The samples of honey were diluted at a volume ratio 1:3 in a concentrated MHB2 medium (CMHB2). The concentrated medium was prepared by dissolution of 22 grams of powdered MHB2 in 750 mL of water instead of 1000 mL as it is required for MHB2 medium. When the honey was added to the concentrated medium in the ratio of 1:3 (*v/v*) the concentration of solid ingredients were exactly the same as in the case of MHB2 medium prepared according to directions (22 grams per 1 L). The obtained honey solutions were filter sterilized with syringe-driven 0.22 μm PES filters (Merck Milipore, city, Ireland). Subsequent series of two-fold dilution of the honey in range 25.0–0.78% (*v/v*) were prepared in a 96-well plate using MHB2 broth. Subsequently, the honey solutions in the wells were inoculated with an equal volume of suspension of bacterial cells, prepared as described previously. The final concentrations of inoculated honeys ranged from 0.39% to 12.5% (*v/v*) (six different concentrations of honeys were tested: 12.5%, 6.25%, 3.12%, 1.56%, 0.78% and 0.39% (*v/v*)). Additionally, positive control of the growth of tested strain and control of the medium sterility was performed.

The plates were incubated 24 h under static conditions at 37 °C. The lowest concentration of honey with no visible bacterial growth was taken as a MIC value. The MIC assay for each tested strain and honey sample was performed three times.

The minimum bactericidal concentrations (MBCs) were assessed by transferring each dilution used for MIC assay on Baird-Parker agar plates using a sterile 48-well microtiter plate replicator. The plates were incubated for 24 h at 37 °C. Concentrations where no growth of the colonies was observed were assigned as MBC.

### 4.5. Activity against Staphylococci Growing in the Form of Biofilm

#### 4.5.1. Biofilm Formation Assay

The assay was performed according to the procedure proposed by Walencka and coworkers [62] with slight modifications. The suspension (OD_600_ = 0.1 in PBS buffer) of *S. aureus* ATCC 25923 cells was prepared as it was described previously and diluted 1:10 (*v/v*) in LB (Luria-Bertani) liquid medium. An aliquot of 200 μL of cells’ suspension were added to the wells of columns 1–7 of vertically set plates. Negative controls were performed with a sterile medium placed in the wells of column 8. The plates were incubated for 24 h at 37 °C in order to allow bacteria to form biofilms.

#### 4.5.2. MBEC Assay

The content of the wells was removed and the wells were washed three times with 200 μL of sterile PBS buffer. 50% (*v/v*) solutions of honey were prepared by dissolution of honey with an equal volume of 2-fold concentrated LB medium and sterilized by filtration (0.22 μm). The 2-fold serial dilutions of honey ranging from 50% to 1.56% or LB medium as positive/negative controls (determination of biofilm growth in the wells that do not contain any growth inhibitors/control of sterility of media) were added to the wells and incubated for 24 h at 37 °C. The MBEC_50_ values of tested honeys were taken as the lowest concentration of honeys that caused at least 50% inhibition of growth of the cells in comparison to the cells growing in the control wells—measured as comparison of ability of living cells to the biotransformation of MTT (3-(4,5-dimethyl-2-thiazolyl)-2,5-diphenyl-2*H*-tetrazolium bromide) to insoluble in water violet formazan crystals [63].

#### 4.5.3. Biofilm MTT-Staining

The MTT assay was performed as described [62,63] with minor modifications. Briefly, after the biofilm formation (according to the procedure presented above) the inoculum was removed and the wells of microplate were washed with 200 μL of sterile PBS buffer. Subsequently, 150 μL of PBS and 50 μL of MTT solution (0.3% in PBS) were added to the wells and mixed. Following 2h incubation at 37 °C in dark, the MTT solution was replaced with 200 μL of DMSO for dissolving of formed formazan crystals. The optical density of the obtained solutions was measured at 540 nm using a Victor^3^ microtiter reader (Perkin Elmer, Waltham, MA, USA).

### 4.6. Time-Kill Assay, Determination of Kinetic of Bactericidal Effect of Honey Against Staphylococci

Time-kill assay was performed for two honeys that revealed the highest anti-staphylococcal activity and manuka honey as a reference. The suspension of approx. cell density 1.5 × 10^6^ CFU/mL of *S. aureus* ATCC 25923 was prepared in MHB2 broth supplemented with honey to the final concentration equal to MBC or 2 × MBC and incubated at 37 °C with shaking. Viable counts of the cells in the suspensions were obtained from 10-fold serial dilutions at 0, 1, 2, 4, 6, 8 and 24 h by plating 10-fold dilution on Baird-Parker agar plates and incubating at 37 °C for 24 h. The number of the cells in the control suspension, without honey addition, was also determined as a control of growth kinetic of *S. aureus* ATCC 25923.

### 4.7. Determination of Hydrogen Peroxide Generation

Hydrogen peroxide concentration in honey was measured according to the modified method described previously by Kwakman and coworkers [5]. Briefly, 25.0% (*w/v*) honey solutions were prepared in deionized water. Samples were incubated at 37 °C for 1 h. 20 μL of diluted honey samples and 67 μL of reagent were added to wells of microtiter plate. The reagent solution consisting of 100 μg/mL of o-dianisidine dihydrochloride and 20 μg/mL of horseradish peroxidase type IV in 10 mM phosphate buffer (pH 6.5). After 5 min of incubation in room temperature, reactions were stopped by addition of 60 μL of 6.0 M H_2_SO_4_. The Absorbance was measured at 540 nm using a Victor^3^ microtiter reader (Perkin Elmer). Concentrations of hydrogen peroxide were calculated using of standard curve the 2-fold serial dilution of H_2_O_2_ standards (550–2.1 μM).

To determine influence of honey components to H_2_O_2_ accumulation 50% (*w/v*) honey solutions were prepared in deionized water and diluted with H_2_O_2_ (200 μM) or water at a ratio 1:1. Samples were incubated at 37 °C for 1 h and concentration of H_2_O_2_ (μM) was measured as described above.

### 4.8. Preliminary Determination of Mechanism of Antimicrobial Activity of Honeys—Heat and Catalase Treatment

Heat treatment and catalase supplementation was performed for preliminary investigation if hydrogen peroxide generation is crucial for antimicrobial activity of tested honeys. To check the influence of elevated temperature to the antimicrobial activity, the 25% (*v/v*) water solutions of selected, the most active honeys were incubated for 10 or 20 min at 40, 60 or 80 °C. After the incubation, the MIC values against *S. aureus* ATCC 25923 were determined according to the procedure described above. The determination of MIC value against *S. aureus* ATCC 25923 was also performed to investigate the influence of catalase on the antibacterial activity of tested honeys samples. It was performed as described above for the procedure of MIC determination, but with the addition of catalase solution to each well in the plates (including positive and negative control) to its final concentration of 250 U/mL.

### 4.9. Total Phenolics Determination

The total content of phenolic compounds was determined using a modified Folin-Ciocalteu method [64]. 0.5 mL of Folin-Ciocalteu reagent was mixed with 100 μL of the honey solution (1:5 *w/v* in ultrapure water) and after 5 min, 3 mL of 100 g/L solution of Na_2_CO_3_ (*w/v*) was added. Following shaking the mixture was made up to a volume of 10 mL with ultrapure water and incubated for 90 min at room temperature. The absorbance at 725 nm was measured against blank in a 10 mm quartz cuvette. Total phenol content was calculated and expressed as milligrams of gallic acid equivalent (GAE) per kilogram, using a calibration curve prepared with fresh gallic acid standard solution (10–500 mg/L). All measurements were performed in triplicate.

### 4.10. Total Antioxidant Activity (FRAP Assay)

The ferric reducing antioxidant assay (FRAP) was performed according to Kuś et al. [64]. 30 μL of the aqueous honey solution (1:5, *w/v*) were dissolved in 2 mL of ferric complex (10 mmol/L TPTZ and 20 mmol/L ferric chloride in acetate buffer (pH 3.6)). Following the 10 min incubation the absorbance at 593 nm was measured in disposable polystyrene cuvettes and the total antioxidant activity was calculated using a calibration curve prepared with ferrous sulfate (0.1–2 mmol/L) as the standard. The results were expressed as millimoles of Fe^2+^ per kilogram of the honey. All measurements were performed in triplicate.

### 4.11. Antiradical Activity (DPPH Assay)

The DPPH assay was performed according to Kuś et al. [64]. 50 μL of the watery honey solution (1:5, *w/v*) were dissolved in 2 mL of 0.04 mmol/L DPPH in methanol. The mixture was incubated for 15 min in dark, at room temperature. Afterwards, the absorbance was measured at 517 nm using disposable polystyrene cuvettes. The data were expressed as Trolox equivalent antioxidant capacity per kilogram of the honey (TEAC, mmol/kg) using calibration curve prepared with Trolox solution (0.05–1.0 mmol/L). All measurements were performed in triplicate.

### 4.12. Determination of Water Content

According to the Polish food law the water content for most types of honeys should not be higher than 20%. Honeys containing more than 20% are usually not microbial stable. The content of water was measured using an ATAGO Hand Refractometer (Atago Co. Ltd., Tokyo, Japan) that enables determination in the range 12–26%.

### 4.13. Determination of pH

The low pH value of honey is also considered as an important factor for microbial potential of this product. It inhibits the growth of endogenous microorganism, but could be also important for antimicrobial activity of honeys used as for example a component of wound dressing materials. The honey samples were dissolved in deionized water to obtain (1:5, *w/v*) solution. The pH of the samples was measured using Hanna Instruments PH200 pH-meter with automatic temperature compensation (Hanna Instruments, Woonsocket, RI, USA) calibrated with pH standards, 4.01 ± 0.02 and 7.01 ± 0.02.

### 4.14. Statistical Analysis

Statistical analyses were performed using GraphPad Prism^®^ 5 (Version 5.01, GraphPad Software, Inc., La Jolla, CA, USA). Correlations were obtained by Pearson’s correlation between the investigated parameters and significance was assessed in two-tailed test at the level of significance *p* < 0.05.

## 5. Conclusions

The results from this study revealed that some honeys produced in Polish apiaries could be used as an alternative agent for treatment of infections caused by staphylococci. Due to the high resistance of staphylococcal biofilm, only undiluted honey or solutions containing at least 25% of the product can be considered for therapeutic purposes. Moreover, only the samples with confirmed high antimicrobial activity could be used for this aim. We also revealed that activity of Polish honeys is hydrogen peroxide-dependent, however phytochemicals are also important for their antimicrobial potential. The results of some of our investigations suggest that the direct mechanism of antimicrobial activity of the product depends on its botanical source.

## Figures and Tables

**Table 1:** Antibacterial activity of Polish honeys against different reference strains of bacteria ^1^.

No.	MIC (%) (*v/v*) against Different Strains of Bacteria									
	*S. aureus* ATCC 25923		*S. aureus* ATCC 29213		*S. epidermidis* ATCC 12228		*E. coli* ATCC 25922		*P. aeruginosa* ATCC 27853	
	MIC	MBC	MIC	MBC	MIC	MBC	MIC	MBC	MIC	MBC
1	12.5	12.5	12.5	12.5	12.5	12.5	>12.5	>12.5	12.5	12.5
2	6.25	>12.5	6.25	>12.5	6.25	>12.5	>12.5	>12.5	12.5	12.5
3	3.125	3.125	6.25	6.25	3.125	3.125	12.5	12.5	6.25	6.25
4	12.5	12.5	12.5	12.5	12.5	12.5	>12.5	>12.5	>12.5	>12.5
5	3.125	6.25	3.125	3.125	3.125	6.25	12.5	12.5	12.5	12.5
6	12.5	>12.5	12.5	12.5	12.5	12.5	>12.5	>12.5	12.5	12.5
7	3.125	12.5	6.25	6.25	3.125	3.125	12.5	12.5	6.25	12.5
8	3.125	3.125	3.125	3.125	3.125	3.125	12.5	12.5	6.25	12.5
9	6.25	6.25	6.25	6.25	6.25	6.25	12.5	12.5	12.5	12.5
10	12.5	12.5	12.5	>12.5	12.5	12.5	>12.5	>12.5	>12.5	>12.5
11	6.25	6.25	6.25	6.25	3.125	6.25	12.5	12.5	12.5	12.5
12	12.5	>12.5	6.25	>12.5	6.25	>12.5	>12.5	>12.5	12.5	12.5
13	12.5	12.5	12.5	12.5	12.5	12.5	12.5	>12.5	12.5	12.5
15	>12.5	>12.5	>12.5	>12.5	12.5	>12.5	>12.5	>12.5	>12.5	>12.5
16	3.125	3.125	3.125	3.125	1.56	1.56	6.25	6.25	6.25	6.25
20	6.25	6.25	6.25	12.5	6.25	6.25	12.5	12.5	12.5	>12.5
21	12.5	12.5	12.5	12.5	12.5	12.5	>12.5	>12.5	12.5	>12.5
22	3.125	6.25	3.125	3.125	3.125	3.125	12.5	12.5	6.25	12.5
23	3.125	6.25	3.125	6.25	3.125	6.25	12.5	12.5	6.25	12.5
24	3.125	6.25	3.125	3.125	3.125	3.125	6.25	6.25	6.25	6.25
25	3.125	6.25	3.125	6.25	3.125	3.125	12.5	12.5	6.25	12.5
27	6.25	6.25	12.5	12.5	6.25	6.25	12.5	12.5	12.5	12.5
28	3.125	3.125	3.125	6.25	3.125	3.125	12.5	12.5	6.25	6.25
30	6.25	6.25	6.25	12.5	6.25	12.5	12.5	>12.5	12.5	12.5
31	3.125	3.125	3.125	3.125	3.125	3.125	12.5	12.5	6.25	6.25
32	12.5	>12.5	>12.5	>12.5	12.5	>12.5	>12.5	>12.5	12.5	>12.5
33	12.5	>12.5	12.5	>12.5	12.5	>12.5	>12.5	>12.5	12.5	>12.5
34	6.25	6.25	6.25	6.25	6.25	6.25	12.5	12.5	12.5	12.5
35	1.56	1.56	1.56	1.56	1.56	1.56	3.125	3.125	3.125	3.125
37	6.25	>12.5	6.25	>12.5	12.5	>12.5	>12.5	>12.5	12.5	>12.5
39	>12.5	>12.5	>12.5	>12.5	12.5	>12.5	>12.5	>12.5	>12.5	>12.5
40	6.25	12.5	6.25	6.25	6.25	6.25	12.5	12.5	12.5	12.5
41	6.25	6.25	6.25	6.25	6.25	6.25	12.5	12.5	12.5	12.5
42	12.5	>12.5	12.5	>12.5	12.5	>12.5	>12.5	>12.5	>12.5	>12.5
43	3.125	6.25	3.125	6.25	3.125	6.25	12.5	>12.5	12.5	12.5
44	6.25	6.25	3.125	3.125	3.125	6.25	12.5	12.5	6.25	12.5
45	3.125	6.25	6.25	6.25	3.125	6.25	12.5	>12.5	12.5	>12.5
46	12.5	>12.5	12.5	>12.5	12.5	>12.5	>12.5	>12.5	12.5	>12.5
47	3.125	6.25	3.125	6.25	3.125	3.125	12.5	12.5	6.25	12.5
49	>12.5	>12.5	>12.5	>12.5	12.5	>12.5	>12.5	>12.5	12.5	>12.5
50	6.25	6.25	6.25	6.25	6.25	6.25	12.5	12.5	12.5	12.5
51	6.25	6.25	6.25	6.25	6.25	6.25	12.5	12.5	12.5	12.5
52	6.25	6.25	6.25	6.25	6.25	6.25	12.5	12.5	12.5	12.5
53	6.25	6.25	6.25	12.5	6.25	>12.5	>12.5	>12.5	12.5	>12.5
54	3.125	6.25	6.25	6.25	3.125	6.25	>12.5	>12.5	12.5	>12.5
55	12.5	12.5	12.5	12.5	12.5	12.5	12.5	12.5	12.5	12.5
57	3.125	3.125	3.125	3.125	3.125	6.25	12.5	12.5	6.25	12.5
60	6.25	6.25	6.25	12.5	6.25	12.5	12.5	>12.5	12.5	>12.5
61	12.5	>12.5	>12.5	>12.5	12.5	>12.5	12.5	>12.5	12.5	>12.5
62	6.25	6.25	6.25	6.25	3.125	3.125	12.5	12.5	12.5	12.5
63	1.56	3.125	1.56	3.125	1.56	1.56	6.25	12.5	6.25	6.25
65	12.5	12.5	12.5	12.5	12.5	12.5	>12.5	>12.5	12.5	>12.5
66	6.25	12.5	12.5	12.5	12.5	12.5	12.5	12.5	12.5	12.5
67	>12.5	>12.5	>12.5	>12.5	12.5	12.5	>12.5	>12.5	>12.5	>12.5
69	6.25	6.25	6.25	6.25	6.25	12.5	12.5	12.5	12.5	12.5
71	6.25	6.25	6.25	6.25	6.25	6.25	12.5	12.5	12.5	12.5
72	6.25	6.25	6.25	6.25	6.25	6.25	>12.5	>12.5	12.5	>12.5
73	6.25	6.25	6.25	6.25	6.25	6.25	12.5	12.5	12.5	12.5
74	6.25	6.25	6.25	6.25	6.25	6.25	>12.5	>12.5	12.5	>12.5
76	3.125	3.125	3.125	3.125	3.125	3.125	6.25	12.5	6.25	6.25
77	3.125	6.25	6.25	12.5	6.25	6.25	12.5	12.5	12.5	12.5
78	6.25	6.25	6.25	6.25	6.25	6.25	12.5	12.5	12.5	12.5
80	12.5	>12.5	>12.5	>12.5	12.5	>12.5	>12.5	>12.5	12.5	>12.5
81	1.56	1.56	1.56	1.56	1.56	1.56	6.25	6.25	3.125	6.25
82	12.5	>12.5	12.5	>12.5	6.25	12.5	>12.5	>12.5	12.5	>12.5
83	1.56	6.25	1.56	1.56	1.56	1.56	6.25	6.25	3.125	6.25
84	12.5	12.5	12.5	12.5	12.5	12.5	>12.5	>12.5	>12.5	>12.5
85	3.125	3.125	6.25	6.25	3.125	3.125	12.5	12.5	6.25	12.5
86	1.56	3.125	1.56	1.56	1.56	1.56	3.125	12.5	3.125	6.25
87	3.125	3.125	3.125	6.25	3.125	3.125	>12.5	>12.5	6.25	>12.5
88	3.125	3.125	3.125	3.125	3.125	3.125	6.25	6.25	6.25	6.25
89	3.125	3.125	3.125	3.125	1.56	3.125	6.25	6.25	6.25	6.25
91	12.5	>12.5	>12.5	>12.5	12.5	>12.5	>12.5	>12.5	12.5	>12.5
92	6.25	6.25	6.25	6.25	6.25	6.25	12.5	12.5	12.5	12.5
94	6.25	6.25	6.25	6.25	6.25	6.25	>12.5	>12.5	12.5	>12.5
95	12.5	>12.5	12.5	12.5	12.5	12.5	>12.5	>12.5	12.5	>12.5
96	6.25	12.5	12.5	12.5	12.5	12.5	12.5	>12.5	12.5	>12.5
98	6.25	12.5	6.25	6.25	6.25	6.25	12.5	12.5	12.5	12.5
99	6.25	12.5	12.5	12.5	6.25	12.5	12.5	>12.5	12.5	12.5
104	12.5	>12.5	12.5	>12.5	12.5	12.5	>12.5	>12.5	>12.5	>12.5
105	3.125	6.25	3.125	3.125	3.125	3.125	6.25	12.5	6.25	6.25
106	12.5	12.5	12.5	>12.5	12.5	12.5	>12.5	>12.5	>12.5	>12.5
107	6.25	6.25	6.25	6.25	6.25	6.25	12.5	>12.5	12.5	12.5
108	3.125	3.125	3.125	3.125	3.125	3.125	6.25	6.25	6.25	6.25
109	6.25	12.5	6.25	6.25	6.25	6.25	12.5	>12.5	12.5	12.5
112	12.5	12.5	12.5	12.5	12.5	12.5	>12.5	>12.5	12.5	>12.5
114	12.5	12.5	12.5	>12.5	12.5	12.5	>12.5	>12.5	>12.5	>12.5
117	6.25	6.25	6.25	6.25	6.25	6.25	12.5	>12.5	12.5	12.5
122	6.25	12.5	12.5	12.5	6.25	12.5	12.5	12.5	12.5	>12.5
123	3.125	6.25	6.25	6.25	6.25	6.25	12.5	12.5	12.5	12.5
124	6.25	6.25	6.25	6.25	6.25	6.25	12.5	12.5	12.5	12.5
125	3.125	6.25	3.125	12.5	3.125	3.125	12.5	12.5	6.25	>12.5
127	6.25	6.25	6.25	6.25	6.25	6.25	12.5	12.5	12.5	12.5
128	3.125	3.125	3.125	3.125	3.125	3.125	6.25	12.5	6.25	6.25
129	12.5	12.5	12.5	12.5	12.5	12.5	12.5	>12.5	12.5	>12.5
130	3.125	3.125	3.125	3.125	3.125	3.125	12.5	12.5	6.25	12.5
131	6.25	6.25	6.25	6.25	6.25	6.25	12.5	12.5	12.5	12.5
132	6.25	>12.5	12.5	>12.5	12.5	12.5	>12.5	>12.5	12.5	>12.5
133	3.125	6.25	3.125	6.25	3.125	3.125	12.5	12.5	6.25	12.5
134	6.25	6.25	6.25	6.25	6.25	6.25	12.5	>12.5	12.5	12.5
135	3.125	3.125	3.125	3.125	3.125	3.125	12.5	12.5	12.5	12.5
137	6.25	6.25	6.25	6.25	3.125	3.125	12.5	12.5	12.5	12.5
138	3.125	6.25	3.125	3.125	3.125	3.125	12.5	12.5	12.5	12.5
139	3.125	3.125	3.125	6.25	3.125	3.125	12.5	12.5	6.25	12.5
140	6.25	6.25	6.25	6.25	6.25	6.25	12.5	>12.5	12.5	12.5
142	>12.5	>12.5	12.5	>12.5	12.5	12.5	12.5	12.5	>12.5	>12.5
143	>12.5	>12.5	>12.5	>12.5	12.5	12.5	>12.5	>12.5	12.5	>12.5
144	3.125	6.25	3.125	3.125	3.125	6.25	6.25	6.25	6.25	12.5

^1^ Results are not presented in the case of honeys which did not reveal any activity against any of the tested strains (MIC and MBC > 12.5% (*v/v*)): 14, 17, 18, 19, 26, 29, 36, 38, , 48, 56, 58, 59, 64, 68, 70, 75, 79, 90, 93, 97, 100, 101, 102, 103, 110, 111, 113, 115, 116, 118, 119, 120, 121, 126, 136, 141).

**Table 2:** Antibacterial activity of the most active Polish honeys against *S. aureus* isolates from subclinical bovine mastitis (SCM) milk samples: SA1, SA9, SA70, SA102, SA103, SA105.

No.	MIC and MBC (%) (*v/v*) against *Staphylococcus aureus* Isolates											
	SA1		SA9		SA70		SA103		SA105		SA102	
	MIC	MBC	MIC	MBC	MIC	MBC	MIC	MBC	MIC	MBC	MIC	MBC
35	1.56	1.56	0.78	3.125	0.78	1.56	1.56	6.25	1.56	6.25	1.56	6.25
24	1.56	1.56	1.56	6.25	1.56	3.125	1.56	>12.5	1.56	12.5	1.56	>12.5
25	3.125	6.25	3.125	12.5	3.125	3.125	3.125	>12.5	3.125	>12.5	3.125	>12.5
86	1.56	1.56	1.56	1.56	1.56	1.56	1.56	6.25	1.56	6.25	1.56	6.25
76	3.125	3.125	3.125	6.25	3.125	3.125	3.125	>12.5	1.56	>12.5	3.125	>12.5
128	3.125	3.125	3.125	6.25	3.125	3.125	3.125	>12.5	3.125	>12.5	3.125	>12.5
105	3.125	3.125	3.125	12.5	3.125	3.125	3.125	>12.5	3.125	>12.5	3.125	12.5
81	1.56	1.56	1.56	3.125	1.56	3.125	1.56	12.5	1.56	6.25	1.56	12.5
108	3.125	3.125	3.125	6.25	3.125	3.125	3.125	12.5	3.125	12.5	3.125	12.5
89	1.56	3.125	1.56	1.56	1.56	3.125	1.56	>12.5	1.56	12.5	1.56	>12.5
139	3.125	3.125	3.125	6.25	3.125	3.125	3.125	>12.5	3.125	>12.5	3.125	>12.5
83	1.56	1.56	1.56	6.25	3.125	3.125	1.56	6.25	1.56	6.25	1.56	6.25

**Table 3:** Antibacterial activity of the most active Polish honeys against *S. aureus* isolates from patients with skin and soft tissue infections (SSTI): Szw35, Szw41, Szw17, Szw48, Szw55, Szw16.

No.	MIC and MBC (%) (*v/v*) against *Staphylococcus aureus* Isolates											
	Szw35		Szw41		Szw17		Szw48		Szw55		Szw16	
	MIC	MBC	MIC	MBC	MIC	MBC	MIC	MBC	MIC	MBC	MIC	MBC
35	1.56	6.25	1.56	6.25	1.56	3.125	1.56	6.25	1.56	6.25	1.56	3.125
24	1.56	12.5	1.56	12.5	1.56	6.25	1.56	12.5	1.56	12.5	1.56	6.25
25	3.125	>12.5	3.125	>12.5	3.125	>12.5	3.125	>12.5	3.125	>12.5	3.125	>12.5
86	1.56	3.125	1.56	6.25	1.56	3.125	1.56	3.125	1.56	6.25	1.56	3.125
76	3.125	>12.5	3.125	12.5	3.125	12.5	3.125	>12.5	3.125	12.5	3.125	12.5
128	3.125	>12.5	3.125	>12.5	3.125	>12.5	3.125	>12.5	3.125	>12.5	3.125	>12.5
105	3.125	>12.5	3.125	>12.5	3.125	12.5	3.125	>12.5	3.125	>12.5	3.125	12.5
81	1.56	6.25	1.56	6.25	1.56	6.25	1.56	6.25	1.56	6.25	1.56	6.25
108	3.125	12.5	3.125	12.5	3.125	12.5	3.125	12.5	3.125	12.5	3.125	12.5
89	3.125	12.5	1.56	12.5	1.56	12.5	3.125	12.5	1.56	12.5	1.56	12.5
139	3.125	>12.5	3.125	>12.5	3.125	>12.5	3.125	>12.5	3.125	>12.5	3.125	>12.5
83	1.56	6.25	1.56	6.25	1.56	6.25	1.56	6.25	1.56	6.25	1.56	6.25

**Table 4:** Activity against staphylococci growing in the form of biofilm.

	MBEC_50_ (%) (*v/v*) against *Staphylococcus aureus* Isolates							
	Szw41	Szw55	Szw48	SA9	SA1	SA70	*S. aureus* ATCC 25923	*S. aureus* ATCC 29213
Sample No. 35	50	50	25	25	50	25	50	25
Sample No. 86	50	50	50	50	50	25	50	25
Manuka honey	50	50	25	25	50	25	50	25

**Table 5:** Preliminary determination of mechanism of honeys activity–heat and catalase treatment.

No.	MIC and MBC (%) (*v/v*) against *Staphylococcus aureus* ATCC 25923							
	40 °C		60 °C		80 °C		Catalase	Untreated Control
	10’	20’	10’	20’	10’	20’		
35	1.56	1.56	6.25	6.25	>12.5	>12.5	12.5	1.56
24	6.25	6.25	>12.5	>12.5	>12.5	>12.5	>12.5	3.125
25	3.125	3.125	6.25	12.5	>12.5	>12.5	>12.5	3.125
86	1.56	1.56	6.25	12.5	>12.5	>12.5	>12.5	1.56
76	3.125	3.125	>12.5	>12.5	>12.5	>12.5	>12.5	3.125
128	3.125	3.125	3.125	12.5	>12.5	>12.5	>12.5	3.125
105	6.25	6.25	12.5	>12.5	>12.5	>12.5	>12.5	3.125
81	1.56	1.56	12.5	>12.5	>12.5	>12.5	>12.5	1.56
108	3.125	3.125	>12.5	>12.5	>12.5	>12.5	>12.5	3.125
89	3.125	3.125	>12.5	>12.5	>12.5	>12.5	>12.5	3.125
139	3.125	3.125	12.5	>12.5	>12.5	>12.5	>12.5	3.125
83	1.56	1.56	6.25	12.5	>12.5	>12.5	>12.5	1.56

**Table 6:** Plant origins of honeys tested in the studies.

Sample Code	Honey Plant Source	*n*	Location
**Professional Beekeepers; *n*** = **95**			
1–58	Multi-floral	*n* = 58	Poland
59–62	Multi-floral collected from forest area	*n* = 4	Poland
63–64	Multi-floral collected from peatland	*n* = 2	Poland
65–68	Rapeseed (*Brassica napus* L.) ^1^	*n* = 4	Poland
69–70	Black locust (*Robinia pseudoacacia* L.) ^1^	*n* = 2	Poland
71–76	Lime tree (*Tilia* spp.) ^1^	*n* = 6	Poland
77–80	Honeydew ^1^	*n* = 4	Poland
81–91	Buckwheat (*Fagopyrum esculentum* Moench) ^1^	*n* = 11	Poland
92	Sunflowers (*Helianthus* spp.) ^1^	*n* = 1	Poland
93	Cowberry. chokeberry (*Vaccinium* spp., *Aronia* spp) ^1^	*n* = 1	Poland
94	Raspberry (*Rubus* spp.) ^1^	*n* = 1	Poland
95	Dandelion (*Taraxacum officinale* F.H. Wigg) ^1^	*n* = 1	Poland
**Organic Grocery Store; *n*** = **22**			
96–97	Multi-floral	*n* = 2	Poland
98	Multi-floral collected from forest area	*n* = 1	Poland
99–100	Rapeseed (*Brassica napus* L.) ^2^	*n* = 2	Poland
101–104	Black locust (*Robinia pseudoacacia* L.) ^2^	*n* = 4	Poland
105–107	Lime tree (*Tilia* spp.) ^2^	*n* = 3	Poland
108–110	Honeydew ^2^	*n* = 3	Poland
111–112	Buckwheat (*Fagopyrum esculentum* Moench) ^2^	*n* = 2	Poland
113	Sunflowers (*Helianthus* spp.) ^2^	*n* = 1	Poland
114	Raspberry (*Rubus* spp.) ^2^	*n* = 1	Poland
115	Dandelion (*Taraxacum officinale* F.H. Wigg) ^2^	*n* = 1	Poland
116	Tansy phacelia (*Phacelia tanacetifolia* Benth.) ^2^	*n* = 1	Poland
117	Clover (*Trifolium* spp.) ^2^	*n* = 1	Poland
**Supermarket; *n*** = **12**			
118–119	Multi-floral	*n* = 2	Poland
120	Rapeseed (*Brassica napus* L.) ^2^	*n* = 1	Poland
121	Black locust (*Robinia pseudoacacia* L.)^2^	*n* = 1	Poland
122	Lime tree (*Tilia* spp.) ^2^	*n* = 1	Poland
123	Lime tree (*Tilia* spp.) ^2^	*n* = 1	Poland. Bulgaria
124–126	Honeydew ^2^	*n* = 3	Poland
127–128	Buckwheat (*Fagopyrum esculentum* Moench) ^2^	*n* = 2	Poland
129	Buckwheat (*Fagopyrum esculentum* Moench) ^2^	*n* = 1	UE countries
**Foreign; *n*** = **15**			
130	Multi-floral	*n* = 1	Italy
131–133	Multi-floral	*n* = 3	Kazakhstan
134–136	Multi-floral	*n* = 3	Ukraine
137	Multi-floral	*n* = 1	Ethiopia
138	Multi-floral	*n* = 1	Crete
139	Multi-floral	*n* = 1	Kazakhstan
t140	Chestnut (*Aesculus* spp.) ^2^	*n* = 1	Italy
141	Eucalyptus (*Eucalyptus* spp.) ^2^	*n* = 1	Italy
142	Orange tree (*Citrus* spp.) ^2^	*n* = 1	Italy
143	Sunflowers (*Helianthus* spp.) ^2^	*n* = 1	Kazakhstan
144	Manuka (*Leptospermum scoparium* J.R. Forst. & G. Forst) ^2^	*n* = 1	New Zealand

^1^ According to the beekepers’ declarations (the botanical source was not verified by pollen analysis). However, honeys derived from some species of plants characterize with some specific features and can be easily recognized. E.g., buckwheat honey is a dark with a very characteristic smell and taste; honey derived from rapeseed is nearly white with a specific consistence and honey produced from lime tree has a specific smell). ^2^ According to the producer declaration.

## References

[1] Samarghandian S., Farkhondeh T., Samini F. (2017). Honey and health: A review of recent clinical research. Pharmacogn. Res..

[2] Hołderna-Kędzia E., Kędzia B. (2002). Miody odmianowe i ich Znaczenie Lecznicze.

[3] Saikaly S.K., Khachemoune A. (2017). Honey and wound healing: An update. Am. J. Clin. Dermatol..

[4] Kwakman P.H., Van den Akker J.P., Guclu A., Aslami H., Binnekade J.M., de Boer L., Boszhard L., Paulus F., Middelhoek P., te Velde A.A. (2008). Medical-grade honey kills antibiotic-resistant bacteria in vitro and eradicates skin colonization. Clin. Infect. Dis..

[5] Kwakman P.H., te Velde A.A., de Boer L., Speijer D., Vandenbroucke-Grauls C.M., Zaat S.A. (2010). How honey kills bacteria. FASEB J..

[6] Anthimidou E., Mossialos D. (2013). Antibacterial activity of Greek and Cypriot honeys against *Staphylococcus aureus* and *Pseudomonas aeruginosa* in comparison to Manuka honey. J. Med. Food.

[7] Brudzynski K., Lannigan R. (2012). Mechanism of honey bacteriostatic action against MRSA and VRE involves hydroxyl radicals generated from honey’s hydrogen peroxide. Front. Microbiol..

[8] Kuncic M.K., Jaklic D., Lapanje A., Gunde-Cimerman N. (2012). Antibacterial and antimycotic activities of Slovenian honeys. Br. J. Biomed. Sci..

[9] Kus P.M., Szweda P., Jerkovic I., Tuberoso C.I. (2016). Activity of Polish unifloral honeys against pathogenic bacteria and its correlation with colour, phenolic content, antioxidant capacity and other parameters. Lett. Appl. Microbiol..

[10] Hammond E.N., Duster M., Musuuza J.S., Safdar N. (2016). Effect of United States Buckwheat honey on antibiotic-resistant hospital acquired pathogens. Pan Afr. Med. J..

[11] Mandal M.D., Mandal S. (2011). Honey: Its medicinal property and antibacterial activity. Asian Pac. J. Trop. Med..

[12] Israili Z.H. (2014). Antimicrobial properties of honey. Am. J. Ther..

[13] Bose B. (1982). Honey or sugar in treatment of infected wounds?. Lancet.

[14] Taormina P.J., Niemira B.A., Beuchat L.R. (2001). Inhibitory activity of honey against foodborne pathogens as influenced by the presence of hydrogen peroxide and level of antioxidant power. Int. J. Food Microbiol..

[15] Mundo M.A., Padilla-Zakour O.I., Worobo R.W. (2004). Growth inhibition of foodborne pathogens and food spoilage organisms by select raw honeys. Int. J. Food Microbiol..

[16] Brudzynski K. (2006). Effect of hydrogen peroxide on antibacterial activities of Canadian honeys. Can. J. Microbiol..

[17] Aljadi A.M., Yusoff K.M. (2003). Isolation and identification of phenolic acids in Malaysian honey with antibacterial properties. Turkish J. Med. Sci..

[18] Bilikova K., Huang S.C., Lin I.P., Simuth J., Peng C.C. (2015). Structure and antimicrobial activity relationship of royalisin, an antimicrobial peptide from royal jelly of *Apis mellifera*. Peptides.

[19] Valachová I., Bučeková M., Majtán J. (2016). Quantification of bee-derived peptide defensin-1 in honey by competitive enzyme-linked immunosorbent assay, a new approach in honey quality control. Czech J. Food Sci..

[20] Kwakman P.H., Zaat S.A. (2012). Antibacterial components of honey. IUBMB Life.

[21] Mavric E., Wittmann S., Barth G., Henle T. (2008). Identification and quantification of methylglyoxal as the dominant antibacterial constituent of manuka (*Leptospermum scoparium*) honeys from New Zealand. Mol. Nutr. Food Res..

[22] Brudzynski K., Miotto D. (2011). Honey melanoidins: Analysis of the compositions of the high molecular weight melanoidins exhibiting radical-scavenging activity. Food Chem..

[23] Lee H., Churey J.J., Worobo R.W. (2008). Antimicrobial activity of bacterial isolates from different floral sources of honey. Int. J. Food Microbiol..

[24] Brudzynski K., Abubaker K., Miotto D. (2012). Unraveling a mechanism of honey antibacterial action: Polyphenol/h(2)o(2)-induced oxidative effect on bacterial cell growth and on DNA degradation. Food Chem..

[25] Irish J., Blair S., Carter D.A. (2011). The antibacterial activity of honey derived from Australian flora. PLoS ONE.

[26] Zainol M.I., Mohd Yusoff K., Mohd Yusof M.Y. (2013). Antibacterial activity of selected Malaysian honey. BMC Complement. Altern. Med..

[27] Brudzynski K., Abubaker K., Wang T. (2012). Powerful bacterial killing by Buckwheat honeys is concentration-dependent, involves complete DNA degradation and requires hydrogen peroxide. Front. Microbiol..

[28] Cao G., Sofic E., Prior R.L. (1997). Antioxidant and prooxidant behavior of flavonoids: Structure-activity relationships. Free Radic. Biol. Med..

[29] Sakihama Y., Cohen M.F., Grace S.C., Yamasaki H. (2002). Plant phenolic antioxidant and prooxidant activities: Phenolics-induced oxidative damage mediated by metals in plants. Toxicology.

[30] Jantakee K., Tragoolpua Y. (2015). Activities of different types of Thai honey on pathogenic bacteria causing skin diseases, tyrosinase enzyme and generating free radicals. Biol. Res..

[31] Ahmed M., Baghdad K., Aissat S., Djebli N. (2016). Colour intensity, polyphenol content and antibacterial capacity of unheated and heat-treated Sahara honey. J. Food Process. Technol..

[32] Brudzynski K., Miotto D., Kim L., Sjaarda C., Maldonado-Alvarez L., Fukś H. (2017). Active macromolecules of honey form colloidal particles essential for honey antibacterial activity and hydrogen peroxide production. Sci. Rep..

[33] Archer N.K., Mazaitis M.J., Costerton J.W., Leid J.G., Powers M.E., Shirtliff M.E. (2011). *Staphylococcus aureus* biofilms: Properties, regulation and roles in human disease. Virulence.

[34] Donlan R.M., Costerton J.W. (2002). Biofilms: Survival mechanisms of clinically relevant microorganisms. Clin. Microbiol. Rev..

[35] Hegazi A.G., Guthami F.M., Gethami A.F., Allah F.M., Saleh A.A., Fouad E.A. (2017). Potential antibacterial activity of some Saudi Arabia honey. Vet. World.

[36] Salonen A., Virjamo V., Tammela P., Fauch L., Julkunen-Tiitto R. (2017). Screening bioactivity and bioactive constituents of Nordic unifloral honeys. Food Chem..

[37] Nishio E.K., Ribeiro J.M., Oliveira A.G., Andrade C.G., Proni E.A., Kobayashi R.K., Nakazato G. (2016). Antibacterial synergic effect of honey from two stingless bees: *Scaptotrigona bipunctata* lepeletier, 1836, and *S. postica* latreille, 1807. Sci. Rep..

[38] Carnwath R., Graham E.M., Reynolds K., Pollock P.J. (2014). The antimicrobial activity of honey against common equine wound bacterial isolates. Vet. J..

[39] Sherlock O., Dolan A., Athman R., Power A., Gethin G., Cowman S., Humphreys H. (2010). Comparison of the antimicrobial activity of Ulmo honey from Chile and Manuka honey against methicillin-resistant *staphylococcus aureus*, *Escherichia coli* and *Pseudomonas aeruginosa*. BMC Complement. Altern. Med..

[40] Alvarez-Suarez J.M., Giampieri F., Brenciani A., Mazzoni L., Gasparrini M., González-Paramás A.M., Santos-Buelga C., Morroni G., Simoni S., Forbes-Hernández T.Y. (2018). *Apis mellifera* vs *Melipona beecheii* Cuban polifloral honeys: A comparison based on their physicochemical parameters, chemical composition and biological properties. LWT Food Sci. Technol..

[41] Ng W.J., Lim M.S. (2015). Anti-staphylococcal activity of Melaleuca honey. Southeast Asian J. Trop. Med. Public Health.

[42] Ronsisvalle S., Lissandrello E., Fuochi V., Petronio Petronio G., Straquadanio C., Crasci L., Panico A., Milito M., Cova A.M., Tempera G. (2017). Antioxidant and antimicrobial properties of *Casteanea sativa Miller* Chestnut honey produced on Mount Etna (Sicily). Nat. Prod. Res..

[43] Moussa A., Noureddine D., Mohamed H.S., Abdelmelek M., Saad A. (2012). Antibacterial activity of various honey types of Algeria against *staphylococcus aureus* and *Streptococcus pyogenes*. Asian Pac. J. Trop. Med..

[44] Al-Waili N., Al-Ghamdi A., Ansari M.J., Al-Attal Y., Salom K. (2012). Synergistic effects of honey and propolis toward drug multi-resistant *Staphylococcus aureus*, *Escherichia coli* and *Candida albicans* isolates in single and polymicrobial cultures. Int. J. Med. Sci..

[45] Ewnetu Y., Lemma W., Birhane N. (2013). Antibacterial effects of *Apis mellifera* and stingless Bees honeys on susceptible and resistant strains of *Escherichia coli*, *Staphylococcus aureus* and *Klebsiella pneumoniae* in Gondar, Northwest Ethiopia. BMC Complement. Altern. Med..

[46] Cooper R. (2016). Honey for wound care in the 21st century. J. Wound Care.

[47] Hussain M.B. (2017). Role of honey in topical and systemic bacterial infections. J. Altern. Complement. Med..

[48] Henatsch D., Wesseling F., Kross K.W., Stokroos R.J. (2016). Honey and beehive products in otorhinolaryngology: A narrative review. Clin. Otolaryngol..

[49] Kwakman P.H., Muller M.C., Binnekade J.M., van den Akker J.P., de Borgie C.A., Schultz M.J., Zaat S.A. (2012). Medical-grade honey does not reduce skin colonization at central venous catheter-insertion sites of critically ill patients: A randomized controlled trial. Crit. Care.

[50] Henatsch D., Nabuurs C.H., van de Goor R.M., Wolffs P.F., Stokroos R.J. (2017). Treatment of recurrent eczematous external otitis with honey eardrops: A proof-of-concept study. Arch. Otolaryngol. Head Neck Surg..

[51] García-Tenesaca M., Navarrete E., Iturralde G., Villacrés Granda I., Tejera E., Beltrán-Ayala P., Giampieri F., Battino M., Alvarez-Suarez J. (2017). Influence of botanical origin and chemical composition on the protective effect against oxidative damage and the capacity to reduce in vitro bacterial biofilms of Monofloral honeys from the Andean region of Ecuador. Int. J. Mol. Sci..

[52] Lu J., Turnbull L., Burke C.M., Liu M., Carter D.A., Schlothauer R.C., Whitchurch C.B., Harry E.J. (2014). Manuka-type honeys can eradicate biofilms produced by *staphylococcus aureus* strains with different biofilm-forming abilities. PeerJ.

[53] Huttunen S., Riihinen K., Kauhanen J., Tikkanen-Kaukanen C. (2013). Antimicrobial activity of different Finnish Monofloral honeys against human pathogenic bacteria. APMIS.

[54] Kędzia B., Hołderna-Kędzia E., Dutkowiak A. (2014). The antibiotic activity of polish Monofloral honeys. Post. Fitoter..

[55] Kretavičius J., Kurtinaitiene B., Račys J., Čekstery V. (2010). Inactivation of glucose oxidase during heat-treatment de-crystallization of honey. Zemdirbyste.

[56] Brudzynski K., Abubaker K., St-Martin L., Castle A. (2011). Re-examining the role of hydrogen peroxide in bacteriostatic and bactericidal activities of honey. Front. Microbiol..

[57] Szczęsna T., Rybak-Chmielewska H. (1997). Antybakteryjne Właściwości Miodu. W: Uzupełniające Zagadnienia Jakości Miodu.

[58] Semkiw P. (2016). Sektor Pszczelarski w Polsce w 2016 Roku.

[59] Sjolund M., Kahlmeter G. (2008). Staphylococci in primary skin and soft tissue infections in a Swedish county. Scand. J. Infect. Dis..

[60] Szweda P., Schielmann M., Frankowska A., Kot B., Zalewska M. (2014). Antibiotic resistance in *Staphylococcus aureus* strains isolated from cows with mastitis in Eastern Poland and analysis of susceptibility of resistant strains to alternative nonantibiotic agents: Lysostaphin, nisin and polymyxin B. J. Vet. Med. Sci..

[61] Szweda P., Toledo A.A. (2017). Antimicrobial activity of honey. Honey Analysis.

[62] Walencka E., Sadowska B., Rozalska S., Hryniewicz W., Rozalska B. (2005). Lysostaphin as a potential therapeutic agent for staphylococcal biofilm eradication. Pol. J. Microbiol..

[63] Kairo S.K., Bedwell J., Tyler P.C., Carter A., Corbel M.J. (1999). Development of a tetrazolium salt assay for rapid determination of viability of BCG vaccines. Vaccine.

[64] Kuś P., Congiu F., Teper D., Sroka Z., Jerkovi C.I., Tuberoso C.I.G. (2013). Antioxidant activity, color characteristics, total phenol content and general HPLC fingerprints of six Polish unifloral honey types. LWT Food Sci. Technol..

